# Modeling cortical dynamics with Wilson-Cowan equations

**DOI:** 10.1186/1471-2202-16-S1-A1

**Published:** 2015-12-18

**Authors:** Jack Cowan

**Affiliations:** 1 Mathematics Department, Neurology Department, and Committee on Computational Neuroscience, University of Chicago, Chicago, IL, USA

## 

Experimental data collected over the last decade indicates that there exist at least two distinct modes of cortical response to stimuli. In mode 1 a low intensity stimulus triggers a wave that propagates at a velocity of about 0.3 m/sec, with an amplitude that decays exponentially. In mode 2 a high intensity stimulus triggers a larger response that remains local, and does not propagate to neighboring regions. Other data indicate that unstimulated or resting cortex exhibits pair correlations between neighboring cells, the amplitudes of which decay slowly with distance, whereas stimulated cortex exhibits pair correlations whose amplitude falls of rapidly with distance. Here we show how the mean-field Wilson-Cowan equations can account precisely for the two modes of cortical response, and how stochastic Wilson-Cowan equations can account for the behavior of the pair correlations. We will present these results after outlining the basic properties of both the mean-field and stochastic equations.

